# Outcomes in critically Ill HIV-infected patients between 1997 and 2020: analysis of the OUTCOMEREA multicenter cohort

**DOI:** 10.1186/s13054-023-04325-9

**Published:** 2023-03-13

**Authors:** Antoine Gaillet, Elie Azoulay, Etienne de Montmollin, Maité Garrouste-Orgeas, Yves Cohen, Claire Dupuis, Carole Schwebel, Jean Reignier, Shidasp Siami, Laurent Argaud, Christophe Adrie, Bruno Mourvillier, Stéphane Ruckly, Jean-Marie Forel, Jean-Francois Timsit

**Affiliations:** 1 Medical Intensive Care Unit, Henri Mondor University Hospital, APHP, 1 Rue Gustave Eiffel, 94010 Créteil Cedex, France; 2 IAME UMR 1137, INSERM, Paris University, 75018 Paris, France; 3 Medical Intensive Care Unit, Saint-Louis University Hospital, APHP, Paris University, 1 Avenue Claude Vellefaux, 75010 Paris, France; 4 Medical Intensive Care Unit, Paris Diderot University/Bichat University Hospital, APHP, Paris, France; 5 Medical Unit, French-British Hospital Institute Levallois-Perret, Levallois-Perret, France; 6 Medical-Surgical Intensive Care Unit, Avicenne University Hospital, Paris Seine Saint-Denis Hospital Network, APHP, Bobigny, France; 7 Medical Intensive Care Unit, CHU Clermont-Ferrand, Clermont-Ferrand, France; 8 Nutrition Humaine Unit, INRAe, CRNH Auvergne, Clermont Auvergne University, 63000 Clermont-Ferrand, France; 9 Medical ICU, Albert Michallon University Hospital, Grenoble 1 University, Grenoble, France; 10 Medical ICU, Nantes University Hospital, Nantes, France; 11 Polyvalent ICU, Sud Essonne Dourdan-Etampes Hospital, Dourdan, France; 12 Medical Intensive Care Unit, Hospices Civils de Lyon, Edouard Herriot University Hospital, Lyon, France; 13 Polyvalent ICU, Delafontaine Hospital, Saint-Denis, France; 14 Medical Intensive Care Unit, Reims University Hospital, Reims, France; 15 Medical ICU, Hôpital Nord University Hospital, Marseille, France

**Keywords:** Human immunodeficiency virus (HIV), Acquired immune deficiency syndrome (AIDS), Antiretroviral therapy (ART), Intensive care unit (ICU), Outcome assessment

## Abstract

**Purpose:**

Despite antiviral therapy (ART), 800,000 deaths still occur yearly and globally due to HIV infection. In parallel with the good virological control and the aging of this population, multiple comorbidities [HIV-associated-non-AIDS (HANA) conditions] may now be observed.

**Methods:**

HIV adult patients hospitalized in intensive care unit (ICU) from all the French region from university and non-university hospital who participate to the OutcomeRea™ database on a voluntary basis over a 24-year period.

**Results:**

Of the 24,298 stays registered, 630 (2.6%) were a first ICU stay for HIV patients. Over time, the mean age and number of comorbidities (diabetes, renal and respiratory history, solid neoplasia) of patients increased. The proportion of HIV diagnosed on ICU admission decreased significantly, while the median duration of HIV disease as well as the percentage of ART-treated patients increased. The distribution of main reasons for admission remained stable over time (acute respiratory distress > shock > coma). We observed a significant drop in the rate of active opportunistic infection on admission, while the rate of active hemopathy (newly diagnosed or relapsed within the last 6 months prior to admission to ICU) qualifying for AIDS increased—nonsignificantly—with a significant increase in the anticancer chemotherapy administration in ICU. Admissions for HANA or non-HIV reasons were stable over time. In multivariate analysis, predictors of 60-day mortality were advanced age, chronic liver disease, past chemotherapy, sepsis-related organ failure assessment score > 4 at admission, hospitalization duration before ICU admission > 24 h, AIDS status, but not the period of admission.

**Conclusion:**

Whereas the profile of ICU-admitted HIV patients has evolved over time (HIV better controlled but more associated comorbidities), mortality risk factors remain stable, including AIDS status.

## Introduction

The Human Immunodeficiency Virus (HIV) pandemic remains a major public health issue with 1.8 million new infections and 800,000 deaths per year [1].

With the development of triple antiretroviral therapy (ART) from 1996 onwards, which allows a better control of HIV, and the improvement of resuscitation techniques (especially ventilation), the prognosis of HIV patients has dramatically improved over the last 25 years [2]. As a result, the HIV population is becoming older, with increasing multiple comorbidities (cirrhosis, chronic obstructive pulmonary disease, renal failure, atherosclerosis, neoplasia), grouped under the term “HIV-Associated Non-Acquired immunodeficiency syndrome (AIDS)” (HANA) conditions. These patients may also be admitted for various symptoms and disease not specific to HIV infection (intoxications, community-acquired co-infections), or related to specific therapies (ART toxicity); as such, they may be present while HIV replication is low or undetectable [2–6].

Although the hospitalization rate of HIV patients has decreased over time (600 vs. 140 per 1000 patient-years in 1999 vs. 2007) [7–9], compared to non-HIV patients, they remain at higher risk (50% excess risk) of admission to intensive care unit (ICU) [6, 10]. Nevertheless, their mortality in ICU in Western countries tends to be similar to that of non-HIV patients [11–19].

The persistence of the pandemic and the phenotypic evolution of HIV patients over time makes relevant to investigate the epidemiology of HIV patients in ICU.

The objectives of this observational study were first to describe the phenotypic characteristics of unselected HIV patients admitted in ICUs from 1997 to 2020, using the French prospective cohort OutcomeRea™, and then to investigate the risk factors for the 60-day mortality after admission to the ICU.

## Methods

The reporting used in this article follows the STROBE recommendations [20].

### Study design and data sources

We conducted an analysis using the prospectively collected data from 1997 to 2020 from all the French region from university and non-university hospital who participate to the OutcomeRea™ database on a voluntary basis (*n* = 23 centers). The OutcomeRea™ database contains data on admission features and diagnosis, daily disease severity, iatrogenic events, nosocomial infections, vital status and decision to forgo life-sustaining therapy (DFLST). Each participating ICU chose to perform sampling by taking either consecutive admissions to randomly selected ICU beds throughout the year or randomly consecutive admissions to all ICU beds over a single month. The data-capture software automatically conducted multiple checks for internal consistency of most of the variables at entry in the database. Queries generated by these checks were resolved with the source ICU before incorporation of the new data into the database. At each participating ICU, data quality was controlled by having a senior physician from another participating ICU check a 2% random sample of the study data. A 1-day coding course is organized annually with the study investigators and contrast research organization monitors. Further details on data collection and quality were described elsewhere [21]. Note that some additional variables were deduced from the database and constructed secondarily (compliance, precariousness).

The OutcomeRea™ database was declared to the “Comité consultatif français de l'informatique pour la recherche en santé” (CCTIRS) et la “Commission française de l'informatique et des libertés” (CNIL, #8,999,262), in accordance with French law, and this study was approved by the ethical committee of the French Society of Intensive Care (SRLF). Waiver for informed consent was granted because the study does not modify patients’ management and the data are anonymously collected.

### Study population

All adult (≥ 18 years) patients diagnosed with HIV or AIDS and registered in the OutcomeRea™ database from 1997 to 2020 were included. The first ICU stay of a patient during the same hospitalization was the only included.

### Definitions

HIV or AIDS, and preexisting chronic organ failures (including respiratory, cardiac, hepatic, renal replacement therapy) were defined according to the Knaus classification [22]. AIDS status was defined as the late stage of HIV infection, i.e., when the number of their CD4 cells fell < 200 cells/mm3 or if an “opportunistic affection” (infectious disease (such as pneumocystis or toxoplasmosis) or hemopathy (such as non-Hodgkin lymphoma or Kaposi's sarcoma) [23]) qualifying for AIDS was developed regardless of their CD4 count. HANA conditions were defined as chronic obstructive pulmonary disease, coronary artery disease, chronic kidney disease, liver cirrhosis and non-AIDS-defining malignancies [18]. The distinction between AIDS, HANA or other classifying conditions was made according to the current classification [23]. An opportunistic infection was considered as a past medical history if it was controlled by > 1 month of effective treatment, whereas a hematological disease required a remission for > 6 months for being considered as past medical history (considered active otherwise). Only CD4 and HIV viral load assays prior to ICU admission and within the last 6 months were considered. A patient was classified as “de novo” HIV if the infection diagnosis was made during the ICU stay or during the prior hospitalization period, otherwise he was classified as “known” HIV. Only “known” HIV patients with a CD4 count > 200/mm^3^ and a negative viral load within the last 6 months were considered “controlled.”

Autonomy was assessed by the Katz scale (ADL) [24]. Disease severity was measured daily by the sepsis-related organ failure assessment (SOFA) [25]. Diagnoses at admission and during the stay were coded using the 10th International Classification of Diseases (ICD). Organ replacement was coded using the Common Classification of Medical Procedures (CCAM).

The ART compliance collected was that reported by the patient in ICU or at the last medical contact when the patient was not able to express himself. Precariousness was based on the few items of the “Agence technique de l'information hospitalière” (ATIH) [26]. Any patient having at least one criterion of precariousness was thus considered precarious.

Finally, DFLST corresponded to a withholding and/or withdrawing treatments aimed at supporting or replacing failing organs (dialysis, vasopressors, mechanical ventilation and cardiopulmonary resuscitation), antibiotics and blood products.

The full study period (1997–2020) was divided into three according to two previously defined dates of interest: 2007, the availability of integrase inhibitors [27]; and 2016, the WHO international recommendation to routinely treat HIV patients regardless of their CD4 count and AIDS-classifying conditions [28, 29].

### Statistical analyses

Characteristics of patients were described as mean (standard deviation), median (interquartile range) or count (percent) for quantitative and qualitative variables, as appropriate. Patient characteristics were compared using the Chi-square test or Fisher’s exact test for categorical variables and the nonparametric Student or Wilcoxon’s rank sum test for continuous variables, as appropriate. The trend tests used to evaluate the periodic evolution were, respectively, a Cochran–Armitage test (or Jonckheere-Terpstra if more than 2 modalities) and an Anova for the categorical and quantitative variables, taking into account the possible center effect.

Factors associated with 60-day mortality were investigated by performing univariate then multiple (with variables yielding *p* ≤ 0.1 in univariate analyses, and/or those of a priori clinical interest according to the literature, included in the model) Cox proportional hazards regression analyses [expressed as hazard ratios (HRs) and 95% confidence intervals (95% CIs)]. Missing data for quantitative and categorical variables were imputed when they represented < 30% of total data by median and mode, respectively. These variables were discarded if there was > 30% missing data (example: last CD4 count). Patients lost to follow-up before day 60 because discharged from hospital were considered alive at day 60. Two-by-two interactions between clinically relevant explanatory variables were tested. Models were stratified by center. Proportional hazards assumption was evaluated using Shoenfeld’s residuals [30].

We used SAS 9.4 (NC, USA) and R software. All tests were two-sided, and p values less than 0.05 were considered significant without taking into account the alpha risk inflation related to multiple comparisons due to the exploratory nature of the analysis.

## Results

**Fig. 1:**
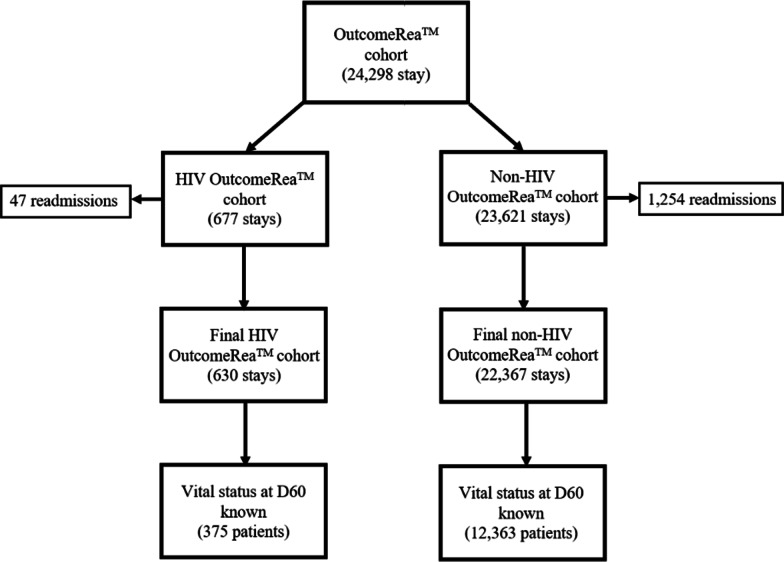
HIV cohort from OutcomeRea™ flowchart. Abbreviations: D60 (day 60 after ICU admission); HIV (Human Immunodeficiency Virus)

Of the 24,298 ICU stays in the OutcomeRea™ cohort, 677 (2.8%) involved HIV patients, including 47 readmissions. The study cohort thus comprises 630 first stays (Fig. 1).

The median age of patients was 46.7 years [38; 55] and 69.8% were men. Approximately 7% of patients had each of the four main comorbidities of Knaus (hepatic, cardiovascular, renal, respiratory); 14.4% of the patients had received prior anticancer chemotherapy. The median SOFA at admission was 5 [2; 8]. The main reasons for admission were acute respiratory distress (35.6%), shock (18.7%) and coma (17.4%); an infection was diagnosed in 54.3% of cases, mostly pneumonia (52.3% of infections) (Table 1).

**Table 1:** General baseline data, overall and by period, of the HIV cohort from OutcomeRea™

	Global (*n* = 630)	1997–2006 (*n* = 215)	2007–2015 (*n* = 336)	2016–2020 (*n* = 79)	*p* value
Age (year)	46.7 [38; 55]	41.8 [36; 51]	48.1 [39; 55]	54.3 [44; 58]	** < 0.001**
Body mass index	21.8 [19; 25]	20.8 [19; 23]	22.7 [20; 25]	23 [20; 27]	** < 0.001**
Katz independence scale	6 [6; 6]	NA	6 [6; 6]	6 [6; 6]	0.991
Sex (male)	440 (69.8%)	143 (66.5%)	242 (72%)	55 (69.6%)	0.350
Diabetes	48 (7.6%)	3 (1.4%)	35 (10.4%)	10 (12.7%)	** < 0.001**
Obesity	34 (5.9%)	4 (2.5%)	18 (5.4%)	12 (15.2%)	** < 0.001**
Substance abuse	79 (13.7%)	30 (18.6%)	40 (12%)	9 (11.4%)	0.464
Precariousness (*n* = 55, 206, 59)	221 (69.1%)	40 (72.7%)	142 (68.9%)	40 (66.1%)	0.446
*Hepatitis*					
B	25 (4%)	6 (2.8%)	18 (5.4%)	1 (1.3%)	0.901
C	66 (10.5%)	13 (6%)	46 (13.7%)	7 (8.9%)	0.098
*Chronic disease (KNAUS)*					
Hepatic	47 (7.5%)	11 (5.1%)	30 (8.9%)	6 (7.6%)	0.229
Cardiovascular	45 (7.1%)	8 (3.7%)	31 (9.2%)	6 (7.6%)	0.066
Renal	41 (6.5%)	7 (3.3%)	27 (8%)	7 (8.9%)	**0.027**
Respiratory	43 (6.8%)	4 (1.9%)	31 (9.2%)	8 (10.1%)	**0.001**
Solid neoplasia	23 (3.6%)	1 (0.5%)	15 (4.5%)	7 (8.9%)	** < 0.001**
Non-AIDS hemopathy	8 (1.5%)	1 (0.7%)	5 (1.6%)	2 (2.7%)	0.134
*Pre-admission immunosuppression (excluding HIV/AIDS)*					
Aplasia	28 (4.4%)	11 (5.1%)	14 (4.2%)	3 (3.8%)	0.560
Corticoid	21 (3.3%)	5 (2.3%)	12 (3.6%)	4 (5.1%)	0.226
Anticancer chemotherapy	91 (14.4%)	26 (12.1%)	56 (16.7%)	9 (11.4%)	0.644
SOT	6 (0.9%)	0	4 (1.2%)	2 (2.5%)	0.037
Other	16 (2.5%)	0	10 (3%)	6 (7.6%)	** < 0.001**
Pre-ICU hospitalization stay (day)	1 [1; 3]	1 [1; 5]	1 [1; 3]	1 [1; 2]	0.324
Medical reason for ICU admission	591 (94%)	199 (92.6%)	318 (94.6%)	74 (94.9%)	0.309
SOFA upon ICU admission	5 [2; 8]	5 [3; 8]	5 [2; 8]	6 [1; 9]	0.668
Main purpose for ICU admission*					0.626
Multivisceral failure	12 (1.9%)	6 (2.8%)	3 (0.9%)	3 (3.8%)	
Septic shock	77 (12.3%)	19 (8.9%)	48 (14.4%)	10 (12.8%)	
Hemorrhagic shock	14 (2.2%)	6 (2.8%)	6 (1.8%)	2 (2.6%)	
Cardiogenic shock	8 (1.3%)	4 (1.9%)	2 (0.6%)	2 (2.6%)	
Shock (other)	18 (2.9%)	6 (2.8%)	10 (3%)	2 (2.6%)	
Acute respiratory distress	223 (35.6%)	85 (39.7%)	106 (31.7%)	32 (41%)	
COPD decompensation	4 (0.6%)	0	4 (1.2%)	0	
Acute renal failure	53 (8.5%)	14 (6.5%)	34 (10.2%)	5 (6.4%)	
Coma	109 (17.4%)	40 (18.7%)	57 (17.1%)	12 (15.4%)	
Continuous monitoring	103 (16.4%)	33 (15.4%)	60 (18%)	10 (12.8%)	
Scheduled surgery	5 (0.8%)	1 (0.5%)	4 (1.2%)	0	
Syndromic diagnosis on admission*					
Infection	342 (54.3%)	131 (60.9%)	166 (49.5%)	45 (57%)	0.133
Bacteremia	29 (4.6%)	15 (7%)	9 (2.7%)	5 (6.3%)	
Pneumonia	179 (28.4%)	80 (37.2%)	74 (22%)	25 (31.6%)	
Meningitis	52 (8.2%)	27 (12.6%)	16 (4.8%)	9 (11.4%)	
Cardiovascular	40 (6.3%)	14 (6.5%)	22 (6.5%)	4 (5.1%)	0.882
Cardiorespiratory arrest	19 (3%)	6 (2.8%)	9 (2.7%)	4 (5.1%)	
Acute lung edema	13 (2.1%)	4 (1.9%)	9 (2.7%)	0	
Myocardial infarction	3 (0.5%)	3 (1.4%)	0	0	
Stroke	5 (0.8%)	1 (0.5%)	4 (1.2%)	0	

Bold indicates the significance of the result (*p* < 0.05)

*AIDS* acquired immunodeficiency syndrome, *COPD* chronic obstructive pulmonary disease, *HIV* human immunodeficiency virus, *SOFA* sepsis-related organ failure assessment, *SOT* solid organ transplant

^*^Only one proposition for each patient

Among these admissions, 199 (37.8%) were related to an AIDS-classifying condition, and 59 (11.2%) to a HANA disease, while 268 (51%) were not directly related to HIV. Overall, 468 (74.3%) patients had a confirmed AIDS, and 232 (51.1%) were not controlled, despite the administration of ART on admission in 313 (58.9%) cases. The median duration of HIV disease prior to admission was 11 years [3; 17], the median last CD4 count and median last viral load were 242/mm^3^ [90; 437] and 2 Log [0; 4.6], respectively (Table 2).

**Table 2:** HIV-related data, overall and by period, of the HIV cohort from OutcomeRea™

	Global (*n* = 630)	1997–2006 (*n* = 215)	2007–2015 (*n* = 336)	2016–2020 (*n* = 79)	*p* value
AIDS	468 (74.3%)	166 (76.7%)	166 (76.7%)	53 (67.1%)	0.123
*HIV status (n* = *454)*					** < 0.001**
De novo	78 (17.2%)	33 (28.4%)	38 (14.2%)	7 (9.8%)	
Known, uncontrolled	232 (51.1%)	57 (49.1%)	151 (56.5%)	24 (33.8%)	
Known, controlled	144 (31.7%)	26 (22.4%)	78 (29.2%)	40 (56.3%)	
Duration of HIV progression (*n* = 411)	11 [3; 17]	5 [2; 13]	12 [5; 17]	18 [7; 25]	** < 0.001**
Last CD4 count (*n* = 282)	242 [90; 437]	223 [104; 400]	228 [81; 400]	324 [130; 539]	**0.014**
Last HIV viral load (*n* = 260)	2 [0; 4.6]	3.1 [0; 5.1]	2.3 [0; 4.6]	0 [0; 2]	**0.004**
Antiretroviral treatment at admission (*n* = 313)	313 (58.9%)	71 (47.6%)	188 (61%)	54 (72%)	** < 0.001**
Therapeutic class					
NRTI	298 (56%)	70 (47%)	175 (56.8%)	53 (70.7%)	
PI	185 (34.8%)	41 (27.5%)	127 (41.2%)	17 (22.7%)	
INI	68 (12.8%)	0	39 (12.7%)	29 (38.7%)	
Pre-resuscitation patient attitude					
Non-compliance (*n* = 370)	137 (37%)	24 (30.4%)	89 (39.4%)	24 (36.9%)	
ART for > 6 months (*n* = 364)	255 (70%)	60 (72.3%)	148 (67.9%)	47 (74.6%)	
History of AIDS-classifying condition*					
Infection	209 (39.3%)	52 (34.9%)	135 (43.8%)	22 (29.3%)	0.896
Pneumocystis	49 (9.2%)	11 (7.4%)	33 (10.7%)	5 (6.7%)	
Tuberculosis	86 (16.2%)	20 (13.4%)	58 (18.8%)	8 (10.7%)	
Toxoplasmosis	38 (7.1%)	2 (1.3%)	31 (10.1%)	5 (6.7%)	
Cytomegalovirus	33 (6.2%)	7 (4.7%)	23 (7.5%)	3 (4%)	
Cryptococcosis	5 (0.9%)	0	4 (1.3%)	1 (1.3%)	
Candidiasis	46 (8.6%)	11 (7.4%)	34 (11%)	1 (1.3%)	
Varicella-Zona virus	77 (14.5%)	18 (12.1%)	51 (16.5%)	8 (10.7%)	
Cryptosporidiosis/Microsporidiosis	11 (2.1%)	5 (3.3%)	6 (1.9%)	0	
Hematologic disease	71 (13.3%)	17 (11.4%)	46 (14.9%)	8 (10.7%)	0.860
Non-Hodgkin's lymphoma	10 (1.9%)	2 (1.3%)	7 (2.3%)	1 (1.3%)	
T lymphoma	1 (0.2%)	0	0	1 (1.3%)	
Kaposi	55 (10.3%)	13 (8.7%)	37 (12%)	5 (6.7%)	
Castelman	21 (3.9%)	8 (5.4%)	12 (3.9%)	1 (1.3%)	
Serous lymphoma	5 (0.9%)	2 (1.3%)	3 (1%)	0	
*Admission by AIDS diagnosis*					
AIDS-classifying conditions*	199 (37.8%)	63 (42.3%)	107 (35.7%)	29 (38.7%)	0.372
Opportunistic infections at admission	135 (25.7%)	54 (36.2%)	63 (20.4%)	18 (24%)	**0.007**
Pneumocystis	49 (9.3%)	21 (14.1%)	19 (6.2%)	9 (12%)	
Tuberculosis	37 (7%)	20 (13.4%)	12 (3.9%)	5 (6.7%)	
Toxoplasmosis	17 (3.2%)	7 (4.7%)	8 (2.6%)	2 (2.7%)	
Cytomegalovirus	40 (7.6%)	11 (7.4%)	22 (7.1%)	7 (9.3%)	
Cryptococcosis	4 (0.8%)	2 (1.3%)	1 (0.3%)	1 (1.3%)	
Candidiasis	31 (5.8%)	10 (6.7%)	17 (5.5%)	4 (5.3%)	
Varicella-Zona virus	10 (1.9%)	1 (0.7%)	7 (2.3%)	2 (2.7%)	
Cryptopsoridiosis	2 (0.4%)	0	2 (0.6%)	1 (1.3%)	
PML	3 (0.6%)	1 (0.7%)	2 (0.6%)	0	
Other	10 (1.9%)	3 (2%)	4 (1.3%)	3 (4%)	
Active hemopathy on admission	146 (27.7%)	29 (19.5%)	99 (32.1%)	18 (24%)	0.154
Non-Hodgkin lymphoma	76 (14.3%)	11 (7.4%)	54 (17.5%)	11 (14.7%)	
T lymphoma	7 (1.3%)	0	6 (1.9%)	1 (1.3%)	
Kaposi	19 (3.6%)	5 (3.3%)	12 (3.9%)	2 (2.7%)	
Castelman	18 (3.4%)	4 (2.7%)	14 (4.5%)	0	
Serous lymphoma	9 (1.7%)	3 (2%)	5 (1.6%)	1 (1.3%)	
Hodgkin lymphoma	16 (3%)	5 (3.3%)	10 (3.2%)	1 (1.3%)	
Other	9 (1.7%)	1 (0.7%)	5 (1.6%)	3 (4%)	
HANA-classifying condition	59 (11.2%)	10 (6.7%)	43 (14.2%)	6 (8%)	0.352
Not associated with HIV	268 (51%)	76 (51%)	152 (49.3%)	40 (53.3%)	0.861

Bold indicates the significance of the result (*p* < 0.05)

AIDS acquired immunodeficiency syndrome, *ART* antiretroviral therapy, *HANA* HIV-associated non-AIDS, *HIV* human immunodeficiency virus, *INI* integrase inhibitor, *PML* progressive multifocal leukoencephalopathy

^*^Several possible proposals per patient

The ICU stay lasted 5 [3; 11] days in median; 45.6% of the patients were mechanically ventilated during their stay, 29% received vasopressors, and 18.9% required renal replacement therapy. Of note, ART was maintained in two cases out of three (68.6%). Finally, 169 (26.8%) patients died before day 60, including 56 (8.9%) DFLST.

### Impact of periods of ICU stay

Over the three periods, there was a significant decrease in the proportion of HIV patients admitted to intensive care (3.2% from 1997 to 2006, 2.6% from 2007 to 2015 and 2.3% from 2016 to 2020, *p* = 0.001 for trend test, adjusted for centers).

As reported in Table 1, the mean age of patients at admission increased over time (44.2 years vs. 51.6 years, *p* < 0.001). There was also an increase over time in the prevalence of most comorbidities, such as diabetes, obesity, solid neoplasia or cardiovascular, renal and respiratory diseases. The SOFA prognostic score at admission to ICU remained stable over time, as did the distribution of the main reasons for admission.

While the proportion of AIDS patients on admission to ICU remained stable over time (*p* = 0.123), the proportion of HIV patients who were controlled on admission almost tripled (22.4% vs. 56.3%), with only 9.8% HIV discovery on admission to ICU in the third period (vs. 28.4% in period 1) (Table 2). Complementarily, there was an increase in the median duration of HIV disease (5 vs. 18 years, *p* < 0.001) and ART coverage at admission (48% vs. 72%, *p* < 0.001) between periods 1 and 3. This was also associated with an improvement in biological markers of disease control, with an increase in median of the last pre-admission CD4 count (223/mm^3^ vs. 324/mm^3^, *p* = 0.014) and a decrease in the median viral load at admission (3.1 Log vs. 0 Log, *p* = 0.004). The rate of opportunistic infection at admission decreased over time (36.2% vs. 24%, *p* = 0.007), while the rate of AIDS-classifying hemopathy increased, although nonsignificantly (19.5% vs. 24%, *p* = 0.154), and the rates of admission for HANA or non-HIV-related were stable (respectively, *p* = 0.352 and *p* = 0.861). Finally, the management of ART evolved over time, with an increase in the rate of ART resumption and initiation between periods 1 and 3 (respectively, 11.8% vs. 40%, *p* = 0.053, and 0% vs. 8.2%, *p* = 0.032).

Regarding organ supplements therapies during ICU stay, the use of mechanical ventilation and renal replacement therapy were stable over time (respectively, from 48.4% to 49.4%, *p* = 0.707, and from 14.9% to 20.2%, *p* = 0.128), while vasopressors were administered significantly more frequently (14.4% vs. 44.3%, *p* < 0.001, with comparable initial SOFA, reason for admission and global amines use over time). Moreover, 12.7% of patients received anticancer chemotherapy during their ICU stay in the third period, compared with 1.4% and 8.3%, respectively, in periods 1 and 2 (*p* < 0.001). Importantly, DFLST rate in ICU was stable over periods (*p* = 0.505) although differences could be seen according to known/controlled HIV status (decrease for de novo HIV (12.1% vs. 0% for period 1 and 3, respectively, *p* = 0.089), increase for known/uncontrolled HIV (3.5% vs. 16.7% for period 1 and 3, respectively, *p* = 0.052) and for known/controlled HIV (11.5% vs. 15% for period 1 and 3, respectively, *p* = 0.050). Finally, the in-ICU and 60-day mortality rates were also stable over time (respectively, 15.8% to 16.5%, *p* = 0.992, and 22.3% to 19%, *p* = 0.382).

### Risk factors for 60-day mortality on ICU admission

Predictors of 60-day mortality are reported in Table 3. Decedents were older, more likely to be men, and had more chronic liver disease and past history of anticancer chemotherapy. Decedents had a higher SOFA score and were more frequently hospitalized for more than 24 h prior to ICU admission. AIDS status, but not the duration of the disease or the last biological activity markers (CD4 count or viral load), was associated with prognosis. We did not find prognostic influence of ART coverage. Finally, 60-day mortality was higher in patients admitted with an active AIDS-classifying hemopathy or HANA, compared with patients admitted to the ICU without HIV involvement.

**Table 3:** Predictors of 60-day after ICU admission mortality in the HIV cohort from OutcomeRea™

	Alive at D60 (*n* = 495)	Dead at D60 (*n *= 135)	Univariate		Multivariate	
			HR, CI 95%	*p* value	HR, CI 95%	*p* value
*Age (years)*						
< 38	126 (25.5%)	29 (21.5%)	**Ref.**	**0.010**	**Ref.**	**0.029**
38 to 54	249 (50.4%)	57 (42.2%)	**0.96 [0.61; 1.50]**		**0.87 [0.54; 1.39]**	
> 54	119 (24.1%)	49 (36.3%)	**1.68 [1.06; 2.67]**		**1.47 [0.91; 2.36]**	
Katz independence scale	6 [6; 6]	6 [6; 6]	0.95 [0.76; 1.19]	0.677		
Sex (male)	333 (67.4%)	107 (79.3%)	**1.70 [1.11; 2.58]**	**0.013**	1.33 [0.85; 2.07]	0.206
Diabetes	37 (7.5%)	11 (8.1%)	1.09 [0.59; 2.02]	0.786		
Obesity	29 (5.9%)	5 (3.7%)	0.67 [0.27; 1.63]	0.375		
*Chronic disease (KNAUS)*						
Hepatic	32 (6.5%)	15 (11.1%)	**1.66 [0.97; 2.85]**	**0.066**	**2.07 [1.15; 3.73]**	**0.015**
Cardiovascular	35 (7.3%)	10 (7.4%)	1.07 [0.56; 2.03]	0.846		
Renal	29 (5.9%)	12 (8.9%)	1.59 [0.87; 2.88]	0.128		
Respiratory	36 (7.3%)	7 (5.2%)	0.77 [0.36; 1.66]	0.507		
Solid neoplasia	17 (3.4%)	6 (4.4%)	1.23 [0.54; 2.80]	0.619		
History of chemotherapy	52 (10.5%)	39 (28.9%)	**3.09 [2.04; 4.68]**	** < 0.001**	**2.48 [1.54; 4.00]**	** < 0.001**
*Inclusion period*						
1(1997–2006)	167 (33.8%)	48 (35.6%)	Ref.	0.929	Ref.	0.578
2(2007–2015)	263 (53.2%)	72 (53.3%)	0.93 [0.63; 1.38]		0.81 [0.54; 1.22]	
3(2016–2020)	64 (13%)	15 (11.1%)	1.00 [0.55; 1.82]		0.82 [0.44; 1.53]	
Medical reason for ICU admission	467 (94.7%)	123 (91.1%)	0.61 [0.33; 1.12]	0.110		
*Main symptom on admission*						
Shock	96 (19.4%)	33 (24.4%)	1.28 [0.79; 2.07]			
Acute respiratory distress	184 (37.2%)	42 (31.2%)	0.93 [0.59; 1.47]			
Coma	82 (16.6%)	27 (20%)	1.31 [0.78; 2.18]			
Other	133 (26.7%)	33 (24.4%)	Ref.	0.392		
SOFA upon ICU admission > 4	262 (53%)	104 (77%)	**2.60 [1.73; 3.88]**	** < 0.001**	**2.35 [1.56; 3.56]**	** < 0.001**
Pre-ICU hospitalization stay > 24 h	172 (34.8%)	72 (53%)	**1.92 [1.36; 2.70]**	** < 0.001**	**1.47 [1.03; 2.11]**	**0.033**
AIDS	355 (71.9%)	112 (83%)	**1.77 [1.12; 2.80]**	**0.014**	**1.79 [1.11; 2.89]**	**0.017**
*HIV status (n* = *528)*						
De novo	63 (15.1%)	15 (13.6%)	Ref.	0.613		
Known, uncontrolled	113 (27%)	26 (23.7%)	1.20 [0.68; 2.12]			
Known, controlled	242 (57.9%)	69 (62.7%)	0.98 [0.52; 1.86]			
Duration of HIV progression > 10 years (*n* = 409)	166 (51.7%)	40 (45.4%)	0.82 [0.54; 1.26]	0.375		
Last CD4 count > 250/mm3 (*n* = 282)	109 (22.1%)	35 (25.9%)	**1.58 [0.92; 2.70]**	**0.096**		
< 50	3 (1.5%)	3 (6.1%)	1.63 [0.80; 3.35]			
50 to 200	60 (30.8%)	20 (40.8%)	1.59 [0.88; 2.85]			
> 200	132 (67.7%)	26 (53.1%)	Ref.			
Last HIV viral load > 2 Log (*n* = 260)	107 (50.7%)	22 (44.9%)	0.78 [0.44; 1.38]	0.394		
ART at ICU admission (*n* = 531)	244 (58%)	69 (62.7%)	1.21 [0.82; 1.79]	0.329		
*History of AIDS-classifying condition*						
Infection	155 (36.8%)	53 (48.2%)	**1.47 [1.00; 2.17]**	**0.048**		
Hematologic disease	54 (12.8%)	20 (18.2%)	1.18 [0.69; 2.01]	0.541		
*Diagnosis admission according to HIV/AIDS*						
AIDS-classifying condition	145 (34.4%)	53 (48.2%)				
Active opportunistic infections	105 (24.9%)	29 (26.4%)	**0.98 [0.65; 1.49]**		1.39 [0.81; 2.39]	
Active hemopathy	96 (22.8%)	50 (45.5%)	**2.59 [1.68; 3.98]**		1.52 [0.94; 2.46]	
HANA	43 (10.2%)	16 (14.5%)	**1.90 [1.06; 3.39]**		1.49 [0.84; 2.64]	
Not associated with HIV	233 (55.3%)	41 (37.3%)	**Ref.**	**0.010**	Ref.	0.203
*ART management in ICU (n* = *316)*						
Suspension	73 (28.1%)	21 (38.2%)	0.98 [0.58; 1.66]	0.942		
Resume	20 (7.7%)	0	**–**	**–**		
Continued	167 (64.5%)	33 (57.9%)	**0.51 [0.32; 0.83]**	**0.006**		
Introduction	22 (8.6%)	4 (7.3%)	0.70 [0.21; 2.27]	0.550		
Mechanical ventilation during the ICU stay	179 (36.2%)	108 (80%)	**5.98 [3.91; 9.14]**	** < 0.001**		
Vasopressor during the ICU stay	113 (22.9%)	70 (51.8%)	**3.08 [2.19; 4.34]**	** < 0.001**		
RRT during the ICU stay	71 (14.4%)	48 (35.6%)	**2.84 [1.98; 4.06]**	** < 0.001**		
Use of anticancer chemotherapy during the ICU stay	25 (5.1%)	16 (11.8%)	**2.22 [1.27; 3.91]**	**0.005**		

Bold indicates the significance of the result (*p* < 0.05)

*AIDS* acquired immunodeficiency syndrome, *ART* antiretroviral therapy, *HANA* HIV-associated non-AIDS, *HIV* human immunodeficiency virus, *ICU* intensive care unit, *RRT* renal replacement therapy, *SOFA* sepsis-related organ failure assessment

**Fig. 2:**
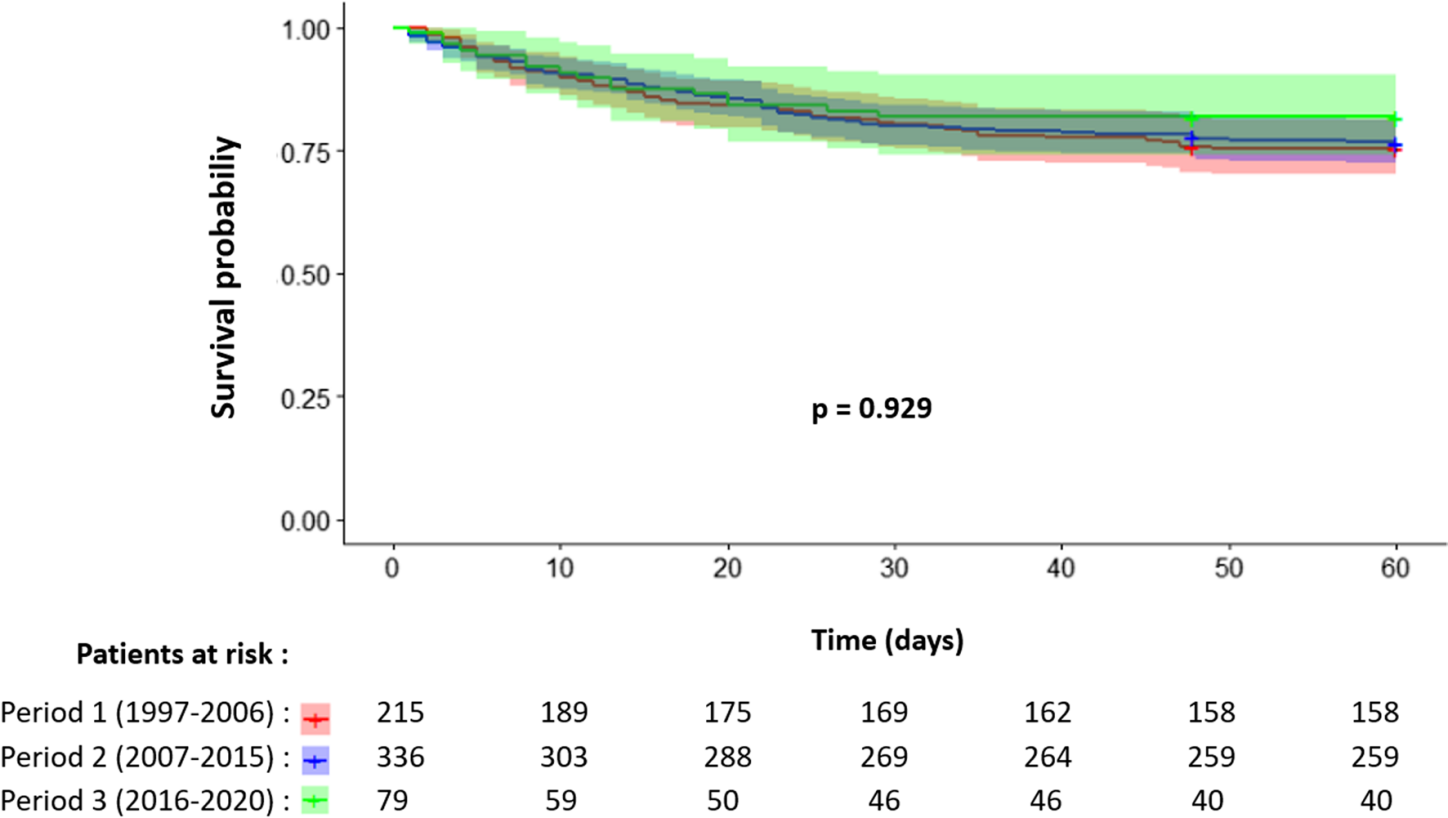
Kaplan–Meier survival curves of HIV patients from the OutcomeRea™ cohort according to the period of care

By multivariate analysis, age > 54 years (HR 1.47 [0.91; 2.36]), chronic liver disease (HR 2.07 [1.15; 3.73], *p* = 0.015), history of anticancer chemotherapy (HR 2.48 [1.54; 4.0], *p* < 0.001), SOFA score > 4 (HR 2.35 [1.56; 3.56], *p* < 0.001), pre-ICU hospitalization duration of stay > 24 h (HR 1.47 [1.03; 2.11], *p* = 0.033) and AIDS status (HR 1.79 [1.11; 2.89], *p* = 0.017) were associated with 60-day mortality. There was a nonsignificant trend toward an increased risk of 60-day mortality for patients admitted for an AIDS-classifying opportunistic infection (HR 1.39 [0.81; 2.39]) or active hemopathy (HR 1.52 [0.94; 2.46]) or for HANA (HR 1.49 [0.84; 2.64]), compared with patients admitted to ICU with no HIV involvement. Of note, the period of care was not associated with the risk of 60-day mortality in univariate (*p* = 0.929) and multivariate (*p* = 0.578) analyses (Fig. 2).

## Discussion

This cohort study confirms and updates epidemiological data on ICU patients with HIV, i.e., an increase in the burden of comorbidities of HIV patients as well as an improvement in the control of the viral infection, and a stability over time of the risk factors for short-term death (more or less directly associated with HIV).

Consistent with the progressive decrease in the level of hospitalization of HIV patients over time [7, 8], the rate of admission to ICU for HIV patients decreased over the study period. The latter is potentially explained by the improvement in HIV control over time, as illustrated by the increase in CD4 count, the decrease in HIV viral load and the increase in ART coverage (up to 70%) at admission, and, as previously reported by Barbier and coll in 2014, the decrease in admissions related to opportunistic infections [6, 10, 18]. Of note, the stability over time and at a high rate (three times higher than described) of the AIDS-classifying hemopathies prevalence is probably related to the center effect induced by a major hematological center (corresponding to 46.3% of the inclusions of the cohort).

The aging of HIV patients, a corollary of their care improvement, is associated with a greater clinical impact of any intoxication, co-infection or of HIV itself (chronic low-level viremia) [2–6]. Consequently, the expected survival benefit of an improved HIV control over time is probably partially offset by the increased burden of comorbidity (mainly respiratory, renal, metabolic diseases and non-AIDS neoplasia) of these patients. Importantly, in accordance with the data in the literature [31, 32] and remaining stable over time, more than half of the patients were admitted to ICU for reason not or indirectly linked to HIV. Moreover, the distribution of the main reasons for admission (in proportion and hierarchy) remains stable over time and overlaps with that of non-HIV patients, as described [31, 32].

Likely indicative of improved specific management of HIV patients in the ICU, the rate of ART resumption and introduction in the ICU increased over time. Although data as to the morbidity-mortality benefit of early HIV treatment is mostly demonstrated in non-critically ill patients [33], there are data to support the same benefit in ICU on short- and long-term prognosis, as outlined in the meta-analysis that Andrade and coll published in 2017 [34]. Meanwhile, the use of organ replacement was stable over time [vasopressor support probably artificially increased because of greater inotrope use during the first period (18.6% vs. 5.1% in period 3, *p* < 0.001, data not shown) [17, 32]. Ultimately, reflecting the paradigm shift in HIV patient care, DFLST rate in ICU has inversely evolved over time, depending on whether HIV was discovered or known (decrease for de novo HIV, increase for known HIV). Knowledge on this subject remains scarce and future studies in view of the phenotypic evolution of the HIV population are warranted [35].

Previous publications on the subject have focused on short-term (ICU/hospital) mortality. After a significant decrease in mortality at the end of the 1990’s, the latest studies reported a stagnation of mortality in intensive care, with values ranging from 16 to 37% according to the region of care [10, 17, 18, 36]. In line with these works, the 60-day ICU mortality rate of the OutcomeRea™ cohort is stable over time. In multivariate analysis, the main risk factors for mortality already described in the past were identified, namely age, history of liver disease or anticancer chemotherapy, AIDS status, severity at admission (estimated by the SOFA score) and duration of the hospital stay before ICU admission. Although there are tendencies for a poorest prognosis of patients with HIV-related reason for hospitalization (mainly active hemopathy), this parameter was no longer associated with prognosis in the multivariate model. When adjusted on all prognostic covariates, the period did not influence the 60-day mortality risk. The persistence over more than 20 years of modifiable risk factors or detectable risk factors invites us to optimize the overall management of HIV patients. Early identification of vulnerable patients would allow an early adaptation of the intensity of care.

The main strengths of this study are its prospective collection and broad and national inclusion period, which provide an accurate evolutionary perspective of the phenotype of HIV patients. In addition, it reassesses and confirms the risk factors for short-term mortality in HIV patients, some of which are avoidable or detectable, and reminds us of the margins for improvement in the management of this population.

The accuracy and completeness of the collection of comorbidities is one of this study limitations. Indeed, neuropsychiatric disorders were not recorded, and the Knaus classification is not very sensitive for the burden of comorbidities. Secondly, the HIV-related biological data could not be fully explored because of a significant lack of collected data (around 50%). Then, the small number of patients in the last period prohibited some subgroup analyses, due to lack of events or power. Finally, the 60-day timepoint for the mortality assessment might have been too early for a reliable picture of the overall risk of death related to ICU admission in these patients.

In conclusion, the phenotype of HIV patients admitted to intensive care is still evolving over time, with an improved control of HIV but an increase in the overall burden of comorbidity. Nevertheless, the medium-term prognosis remains stable over time. Several questions remain unanswered; the long-term post-resuscitation outcome, particularly in terms of quality of life, and the management of antiretroviral drugs require further explorations.

## Data Availability

The data that support the findings of this study are available on request from the corresponding author [AG].
